# Intracytoplasmic maturation of the human immunodeficiency virus type 1 reverse transcription complexes determines their capacity to integrate into chromatin

**DOI:** 10.1186/1742-4690-3-4

**Published:** 2006-01-12

**Authors:** Sergey Iordanskiy, Reem Berro, Maria Altieri, Fatah Kashanchi, Michael Bukrinsky

**Affiliations:** 1 Department of Microbiology, Immunology and Tropical Medicine, The George Washington University, 2300 I St. N.W., Washington, DC 20037, USA; 2 Department of Molecular Virology, The D.I. Ivanovsky Institute of Virology, 16 Gamaleya St., Moscow 123098, Russia; 3 Department of Biochemistry and Molecular Biology, The George Washington University, 2300 I St. N.W., Washington, DC 20037, USA

## Abstract

**Background:**

The early events of the HIV-1 life cycle include entry of the viral core into target cell, assembly of the reverse transcription complex (RTCs) performing reverse transcription, its transformation into integration-competent complexes called pre-integration complexes (PICs), trafficking of complexes into the nucleus, and finally integration of the viral DNA into chromatin. Molecular details and temporal organization of these processes remain among the least investigated and most controversial problems in the biology of HIV.

**Results:**

To quantitatively evaluate maturation and nuclear translocation of the HIV-1 RTCs, nucleoprotein complexes isolated from the nucleus (nRTC) and cytoplasm (cRTC) of HeLa cells infected with MLV Env-pseudotyped HIV-1 were analyzed by real-time PCR. While most complexes completed reverse transcription in the cytoplasm, some got into the nucleus before completing DNA synthesis. The HIV-specific RNA complexes could get into the nucleus when reverse transcription was blocked by reverse transcriptase inhibitor, although nuclear import of RNA complexes was less efficient than of DNA-containing RTCs. Analysis of the RTC nuclear import in synchronized cells infected in the G2/M phase of the cell cycle showed enrichment in the nuclei of RTCs containing incomplete HIV-1 DNA compared to non-synchronized cells, where RTCs with complete reverse transcripts prevailed. Immunoprecipitation assays identified viral proteins IN, Vpr, MA, and cellular Ini1 and PML associated with both cRTCs and nRTCs, whereas CA was detected only in cRTCs and RT was diminished in nRTCs. Cytoplasmic maturation of the complexes was associated with increased immunoreactivity with anti-Vpr and anti-IN antibodies, and decreased reactivity with antibodies to RT. Both cRTCs and nRTCs carried out endogenous reverse transcription reaction *in vitro*. In contrast to cRTCs, *in vitro *completion of reverse transcription in nRTCs did not increase their integration into chromatin.

**Conclusion:**

These results suggest that RTC maturation occurs predominantly in the cytoplasm. Immature RTCs containing RT and incomplete DNA can translocate into the nucleus during mitosis and complete reverse transcription, but are defective for integration.

## Background

The early events of the HIV-1 life cycle include entry of the viral core into target cell, assembly of the reverse transcription complexes (RTCs), reverse transcription of the viral genome and transformation of RTCs into integration-competent complexes called pre-integration complexes (PICs) [1], trafficking of PICs into the nucleus, and finally integration of the viral DNA into chromatin (reviewed in ref [2]. Molecular details and temporal organization of these processes remain among the least investigated and most controversial problems in the biology of HIV. For example, reverse transcription is generally completed in 8 to 12 h, whereas virus-specific DNA can be detected in the nuclei of infected cells as early as 4 h post-infection [3]. This and the finding that nuclear complexes may contain RT [4] question the retrovirology dogma that reverse transcription completes in the cytoplasm and suggest that HIV-1 RTC maturation may occur after translocation into the nucleus.

HIV-1 nucleoprotein complexes isolated from the cytoplasm of infected cells (cRTCs) contain reverse-transcriptase (RT), integrase (IN), matrix protein (MA) and Vpr [4-6] The capsid protein (CA) was detected in virus-specific complexes early after infection, but it was absent in cRTCs analyzed at later time points and in nuclear RTCs (nRTCs) [4,7] The composition of the HIV-1 nPICs is still unclear. Early studies suggested that IN alone is sufficient for efficient integration, at least *in vitro *[1,8]. Later, viral proteins MA and Vpr, and even RT were identified in the nuclear compartment in detectable amounts [4,9,10]. In addition, certain cellular proteins involved in chromatin organization and remodeling, such as the high mobility group protein HMGA [11,12], SWI/SNF component Ini1 and PML [13], associate with the HIV-1 RTC during its migration from the cytoplasm into the nucleus and may contribute to integration or some pre-integration event in the nucleus, such as regulating intranuclear movements of RTC or modifying the chromatin at the site of integration. It becomes clear that the RTC undergoes substantial reorganization coinciding with its migration from the cytoplasm into the nucleus. It should be noted here that only a small proportion of RTCs produced in each cell finally integrates and gives rise to progeny virions, whereas biochemical studies deal with a bulk of virus-specific complexes. Nevertheless, most likely all the complexes that initiated reverse transcription follow the same steps of maturation, though many of them either arrest at some stage before completion of reverse transcription or complete reverse transcription but do not integrate because of intranuclear restrictions. Thus, in this study, we focused on comparative analysis of protein composition, reverse transcription and integrative capacity of the cytoplasmic and nuclear complexes of HIV-1. We demonstrate that RTCs can be translocated into the nucleus at different stages of reverse transcription and that population of nuclear complexes is heterogeneous, although nuclear translocation of complexes in which reverse transcription had been blocked is less efficient than of RTCs containing full-length HIV-1 DNA. Nuclear import of the HIV-specific nucleoprotein complexes is associated with qualitative and quantitative changes in their protein content. Apparently, these changes correlate with translocation of RTCs through the nuclear pore complex (NPC), because passing of the cells through mitosis favored accumulation in the nucleus of immature RTCs containing incomplete DNA. These RTCs appear to be impaired in integration capacity even after completion of reverse transcription.

## Results and Discussion

### Analysis of HIV-1 reverse-transcription complexes during first hours of infection

**Figure 1:**
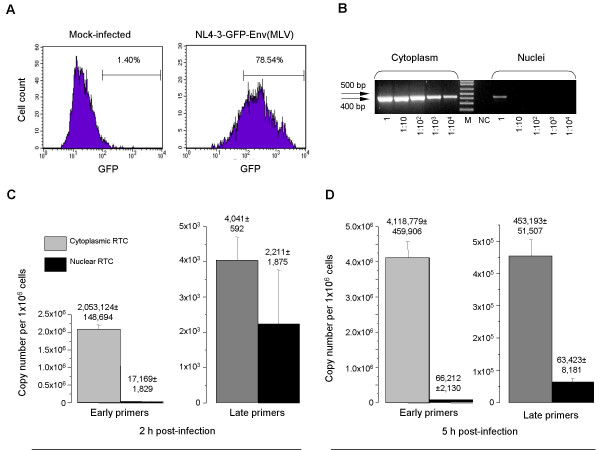
**Analysis of nucleo-cytoplasmic distribution of HIV-1 RTCs**.HeLa cells were spinoculated with MLV Env-pseudotyped NL4-3 or NL4-3-GFP HIV-1. A. HeLa cells infected with GFP-expressing HIV-1 were analyzed by FACS 48 h after infection. Percentage of GFP-positive cells was counted using CellQuest software. B. PCR analysis of the purity of nuclear extracts. Cytoplasmic and nuclear extracts were prepared from the same number of cells (1 × 10^6^) and total DNA was isolated. Undiluted and diluted (1:10, 1:10^2^, 1:10^3^, and 1:10^4^) DNA samples were analyzed by PCR using primers specific for mitochondrial DNA. M – DNA molecular size marker, NC – negative control (H_2_O). C,D. Real-time PCR analysis of nuclear and cytoplasmic RTCs. DNA isolated from cytoplasmic and nuclear RTCs 2 h (C) and 5 h (D) after spinoculation was analyzed in triplicate with primers specific for early or late HIV-1 DNA using SYBR Green qPCR. Serial dilutions of DNA from 8E5 cells were used as quantitative standards. Results are presented as mean ± SD.

Nuclear and cytoplasmic RTCs were purified from HeLa cells which were infected with DNase I-treated MLV Env-pseudotyped HIV-1 by spinoculation [14]. This procedure allowed infection of 70–80% of the cells, as shown using the GFP-expressing NL4-3 HIV-1 (Fig. 1A), which was generated by transfecting HEK 293T cells with NL43GFP11 molecular clone [15]. Of note, infection of HeLa CD4+ cells with non-pseudotyped HIV-1 produced 10-fold lower level of infection (data not shown). Therefore, the use of pseudotyped HIV-1 construct was necessary for high efficiency of infection required for our analysis, as we failed to obtain consistent results with the wild-type HIV-1. In previous studies [3], VSV-G pseudotyping was used to increase efficiency of infection, however, this envelope mediates entry via endocytosis, whereas the MLV envelope mediates fusion at the plasma membrane [16], similar to the entry pathway used in normal HIV infection process. Cytoplasmic contamination of the nuclear fractions was negligible and did not exceed 0.1%, as illustrated by PCR amplification of mitochondrial DNA from cytoplasmic and nuclear extracts (Fig. 1B).

Analysis of cRTCs 2 h post-infection showed substantially more complexes with early ("strong-stop") DNA than with late reverse transcription products (2.05 versus 0.004 copies per cell, respectively) (Fig. 1C). The number of complexes carrying early reverse transcription product increased two-fold at 5 h post-infection (compare panels C and D in Fig. 1), suggesting that many virions began reverse transcription later than two hours post-entry. The ratio of complexes carrying early and late RT products was 500:1 after 2 h (Fig. 1C), and 10:1 after 5 h of infection (Fig. 1D) (i.e., the proportion of late DNA-containing cytoplasmic complexes increased fifty-fold in 3 hours). Nevertheless, at least 90% of complexes in the cytoplasm did not complete reverse transcription during first 5 h of infection, as late primers recognized only about 10% of RTCs recognized by early primers (Fig. 1D). The observed ratios correlate well with previously published data [17,18]. obtained using different approaches, thus validating our experimental system. A much higher number of complexes per cell in our analysis than in previous studies was likely due to the method of infection, which allows to synchronously infect at least 75% of the cells (Fig. 1A). Thus, the number of cytoplasmic HIV-1 complexes initiating reverse transcription increases approximately 2-fold (from 2 to approximately 4 complexes per cell) during the period from 2 h to 5 h after infection.

Comparative analysis of strong-stop HIV-1 cDNA (an early RT product) in cytoplasmic and nuclear RTCs at 2 h post-infection revealed the ratio of cytoplasmic to nuclear complexes as 120:1, which decreased two-fold (to 60:1) during subsequent 3 h incubation (Fig. 1C,D). This decrease likely reflects the process of nuclear translocation of the cytoplasmic complexes. It should be noted that proteasomal degradation of the early HIV-1 infection intermediates described in [19-21] is unlikely to play significant role in our experimental conditions, as early viral DNA increased two-fold from 2 h to 5 h post-infection and a substantial amount of early RTCs carried on to synthesize late DNA (Fig. 1C,D). Proportion of RTCs containing late reverse transcription products in the total population of complexes (estimated by measuring strong-stop DNA copies) increased hundred-fold from 2 h to 5 h post-infection (due to ongoing reverse transcription), whereas proportion of nRTCs containing late HIV-1 DNA increased only thirty-fold (panels C and D in Fig. 1). Furthermore, for the first two hours after infection, RTCs in the nuclear compartment carried predominantly the early HIV-1 reverse transcription products (17,169 copies of early DNA and 2,211 copies of late DNA, Fig. 1C), whereas at 5 h post-infection more than 95% of nRTCs contained late reverse transcription products (66,212 copies of early DNA and 63,423 copies of late DNA, Fig. 1D).

These results demonstrate that proportion of RTCs carryind late reverse transcripts increases in both cytoplasmic and nuclear compartments during the course of infection. Since the relative growth of these complexes was higher in the nucleus than in the cytoplasm, we next investigated whether this phenomenon was a result of selective nuclear import of RTCs containing full-length reverse transcription product (mature RTCs).

**Figure 2:**
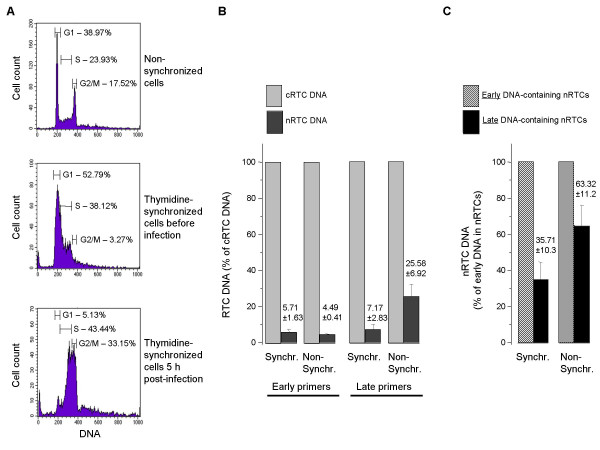
**Quantitative analysis of nuclear translocation of HIV-1 RTCs in synchronized cells**. A. Cell cycle distribution of control, non-synchronized HeLa cells (upper panel), and cells pre-treated with 2 mM thymidine was measured by flow cytometric analysis before spinoculation (middle panel) and 5 h after spinoculation (lower panel). Percentage of cells at different phases of the cell cycle was counted using CellQuest software. B,C. Nuclear translocation of HIV-1 RTCs. HIV-1 DNA was purified from cytoplasmic and nuclear HIV-1 complexes 5 h after infection of synchronized and non-synchronized HeLa cells. Triplicate samples were analyzed by real-time PCR with primers specific for early and late HIV-1 DNA by measuring SYBR Green fluorescence. Values are means ± SD. Panel B shows percentage of nRTC DNA relative to DNA from cRTCs. Panel C represents percentage of late DNA from nRTCs relative to early nRTC DNA.

Both immature and mature HIV-1 RTCs can get into the nucleus during mitosis, as this mechanism is non-discriminative and is used by many retroviruses [22-24] In non-synchronized cultures, as is the case with HeLa cells in our experiments, the changes in the number of cells going through mitosis at different time points may influence the distribution of cytoplasmic and nuclear RTCs. To eliminate this complication, we quantitatively analyzed nuclear import of RTCs in synchronized cells. This approach was selected over analysis of infection in growth-arrested cells because of apoptotic activity (which may significantly and unpredictably affect results of analysis) of practically all cell cycle-arresting agents. After treatment with thymidine, HeLa cells were synchronized in the G1/S phase (90.9% of cell population, middle panel in Fig. 2A). Cells were infected with MLV-pseudotyped HIV-1, incubated in fresh medium for 5 h and analyzed by flow cytometry for cell cycle distribution. This analysis revealed that one third (33%) of synchronized cells shifted to G2/M phase of the cell cycle (low panel in Fig. 2A), whereas in non-synchronized culture percentage of dividing cells did not exceed 17% (upper panel, Fig. 2A). Real-time PCR analysis of cytoplasmic and nuclear RTCs showed a slight increase in the proportion of nuclear RTCs (judged by early DNA) in synchronized (5.71%) compared to non-synchronized cells (4.49%, Fig. 2B). However, the proportion of nuclear late DNA-containing RTCs was significantly higher in non-synchronized cells (25.58% vs 7.17%, Fig. 2B), suggesting that nuclear import in non-synchronized cells favors RTCs with full-length DNA. In synchronized, actively dividing cells, late DNA-containing RTCs constituted one third (35.71%) of the total nRTC population, while in non-synchronized cells their proportion reached two thirds (63.32%) (Fig. 2C). It should be noted that our analysis likely underestimates the amount of nRTCs in synchronized cells, as 33% of these cells are in G2/M phase (Fig. 2A) and may lack the nuclei. However, accounting for these cells would not significantly change the cytoplasm/nuclear ratio of early and late DNA-containing RTCs, as nuclear RTCs constitute less than 10% in synchronized cells (Fig. 2B). These data show that in synchronously dividing cells, the ratio of nRTCs carrying early and late reverse transcription products is similar to that in cRTCs, whereas in normal, non-synchronized cell population the nuclear fraction is clearly enriched in RTCs containing late HIV-1 DNA. This finding suggests that most of the early DNA-containing RTCs get into the nuclear compartment during mitosis. RTCs carrying complete HIV-1 DNA seem to have an advantage in translocation through the NPC.

**Figure 3:**
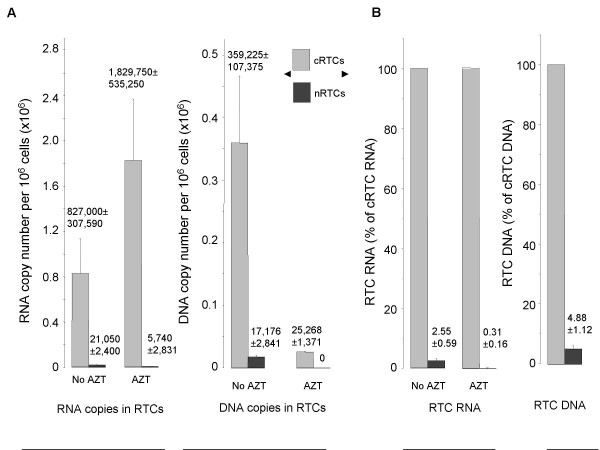
**Nuclear translocation of RNA and DNA containing HIV-1 PICs**. DNA and RNA were purified from cytoplasmic and nuclear HIV-1 complexes 5 h after infection of HeLa cells in the presence or absence of AZT (3 μM). Triplicate samples were analyzed by real-time PCR with primers specific for late HIV-1 DNA by measuring SYBR Green fluorescence. Results are presented as mean ± SD. A. Absolute values of nuclear and cytoplasmic HIV-1 DNA and RNA in RTCs. B. Percentage of nuclear RNA or DNA relative to cytoplasmic RNA or DNA, respectively.

To further test this idea, we analyzed the translocation from the cytoplasm to the nucleus of RNA-containing complexes in which reverse transcription was artificially inhibited. Non-synchronized HIV-infected HeLa cells were treated with AZT (3 μM) to block reverse transcription. Cytoplasmic and nuclear HIV-1 complexes were isolated from AZT-treated and untreated cell extracts 5 h post-infection, and RNA or DNA was purified and analyzed by real-time PCR using primers specific for late HIV-1 reverse transcripts. As shown in Figures 3, the efficiency of the nuclear import (as judged by the percentage of nuclear versus cytoplasmic RTCs) of DNA-containing complexes (4.88%, panel B) was about two-fold higher compared to RNA-containing complexes (2.55%, panel B). AZT treatment increased the number of RNA-containing complexes in the cytoplasm by 2.2-fold (Fig. 3A), however, only 0.31% of these complexes got into the nucleus, whereas almost 5% of DNA-containing RTCs translocated into the nucleus (Fig. 3B). Lower efficiency of nuclear translocation of HIV-1 complexes incapable of performing reverse transcription may be due to conformational restraints (e.g., excessive size of the complexes) or to the lack or inaccessibility of determinants required for efficient nuclear import (e.g., DNA flap [25]). Likely, most of these immature particles get into the nuclear compartment during mitosis. This conclusion is consistent with a dramatic decrease of nuclear import of RNA-containing complexes after AZT treatment (from 2.5% to 0.3% in Fig. 3B), which can be explained in part by AZT-induced arrest in the S phase of cell cycle of the treated cells [26].

Taken together, presented results suggest that HIV-1 RTCs can get into the nucleus at the time of mitosis in a non-selective manner, or they can translocate through the NPC. The latter pathway appears to be selective for RTCs which have completed reverse transcription.

### Protein composition of RTCs

**Figure 4:**
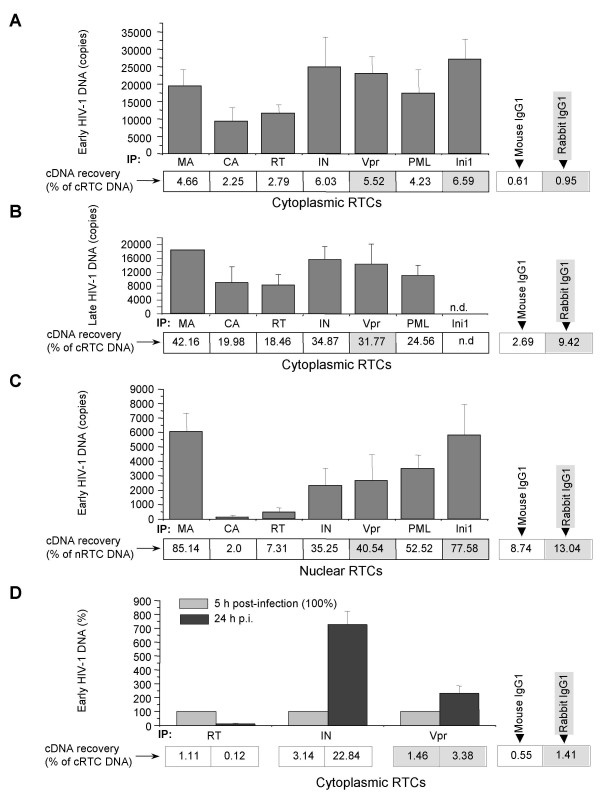
**Analysis of protein composition of cytoplasmic and nuclear RTCs**. cRTCs and nRTCs purified 5 h after infection were immunoprecipitated using the indicated antibodies and Protein G Sepharose. DNA was isolated from immune complexes and analyzed by real-time PCR as in Fig. 1. DNA recovered in immunoprecipitated RTCs as percentage of total HIV-1 DNA detected in the cRTCs is indicated under the histogram columns. DNA recovery for isotype control antibodies is shown on the right. DNA recovery for mouse mAb is shown in open boxes, for rabbit polyclonal antibodies – in shaded boxes. A,B. Immunoprecipitated cRTCs were analyzed using primers specific for early (A) and late (B) reverse transcription products. N.d. – not done. Results are mean ± SD of triplicate determinations, except for late DNA analysis of anti-MA-precipitated complexes, which was done only once. One representative experiment out of 4 performed is shown. C. Experiment was performed as in A, except that nRTCs were analyzed. Low sensitivity of primers specific for late HIV-1 DNA precluded their use for analysis of nRTCs. Results are mean ± SD of triplicate determinations. One representative experiment out of 4 performed is shown. D. Temporal analysis of cRTCs. Results are mean ± SD of triplicate determinations. One representative experiment out of 3 performed is shown.

Protein composition of cytoplasmic and nuclear complexes of HIV-1 was analyzed 5 h post-infection using immunoprecipitation (IP) followed by real-time PCR analysis of HIV-1 DNA as described in the Method section. Because of a lower sensitivity of PCR with primers specific for late cDNA than early cDNA, we could not use late primers for analysis of immune precipitates of nRTCs. It should be noted that the rate of cDNA recovery (ratio of cDNA in immunoprecipitated RTCs to total RTC cDNA) in immunoprecipitates of cytoplasmic RTCs obtained with primers specific for early HIV-1 DNA was lower, than with primers, specific for late DNA (Fig. 4A,B), likely due to the presence of a large number of internalized virions (intact or only partially uncoated) and products of virion degradation in the cytoplasm. Analysis of cRTCs immunoprecipitated with anti-Vpr and anti-IN antibodies 24 h after infection showed a two-fold and seven-fold increase, respectively, in the level of HIV-1 DNA recovery compared to complexes analyzed 5 h after infection, whereas recovery of HIV-1 DNA in complexes immunoprecipitated with anti-RT antibody decreased almost 10-fold (from 1.11% to 0.12%, Fig. 4D). This result suggests that protein composition or conformation of cytoplasmic complexes changes during the process of their maturation. The data obtained using late DNA-specific primers (Fig. 4B) indicate higher values of DNA recovery, which may reflect higher accessibility of proteins to antibodies in RTCs completing their maturation.

Our analysis demonstrates that most proteins identified in cRTCs were also present in nRTCs (Fig. 4C). It is unlikely that this result was due to cytoplasmic contamination of the nuclear fractions, as nuclear RTCs were impoverished in RT, and minimal quantity of mitochondrial DNA could be detected in the nuclear fractions (Fig. 1B). Analysis of nRTCs immunoprecipitated with antibody to CA, which has been previously found in early intermediates of HIV-1 infection [7], revealed only negligible levels of early reverse transcription complexes (Fig. 4C). However, some nRTCs could be immunoprecipitated with anti-RT antibody (Fig. 4C). This finding suggests that some RTCs may complete reverse transcription in the nucleus. Low levels of RT-containing complexes in nRTC population are consistent with a time-dependent decrease in RT representation in cRTCs (Fig. 4D). These data show that nRTCs appear as a heterogeneous population of particles, containing complexes at different stages of reverse transcription and characterized by different protein composition. This heterogeneity in protein content may explain the heterogeneity in buoyant density reported by Fassati and Goff [3].

### Endogenous reverse transcription (ERT) in RTCs

**Figure 5:**
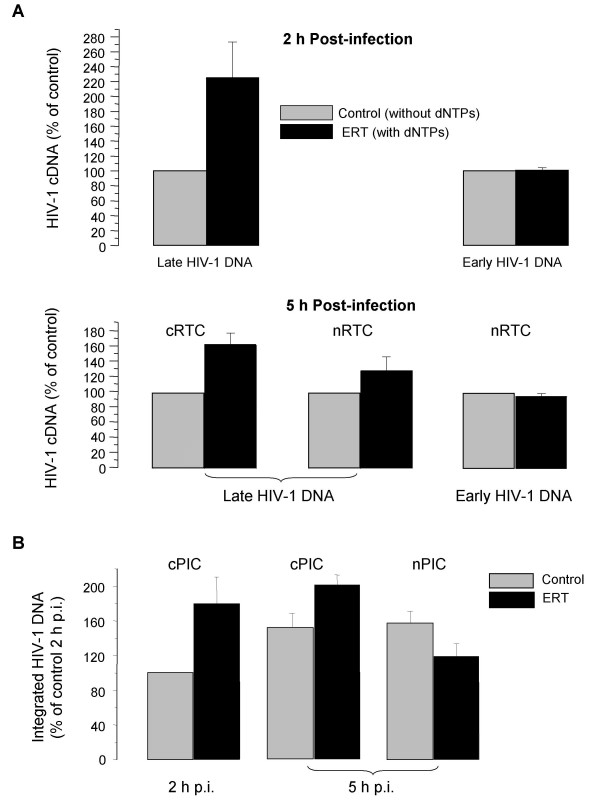
**Quantitative PCR analysis of ERT activity and integration of cytoplasmic and nuclear RTCs**. A. ERT activity of cRTCs and nRTCs isolated 2 h and 5 h post-infection. cRTCs and nRTCs were normalized according to strong-stop (early) HIV-1 DNA content measured by real-time PCR. ERT reaction was performed in duplicate as described in the text. HIV-1 DNA was quantified by real-time PCR. HIV-1 DNA in RTCs incubated without dNTPs (control) was taken as 100%. Results are presented as mean ± SE. B. Quantitative PCR analysis of PIC integration into chromatin. cPICs and nPICs after the ERT reaction performed with or without (control) dNTPs were incubated in triplicate with chromatin samples. DNA was purified and analyzed by *Alu*-LTR-based real-time nested PCR [29]. Integration efficiency was evaluated relative to integration of cPIC isolated 2 h p.i. Results are presented as mean ± SD.

Since RT was found in both cytoplasmic and nuclear complexes, we analyzed their capacity to perform endogenous reverse transcription (ERT). Cytoplasmic complexes isolated at 2 h post-infection showed a 2.4-fold increase in the number of late reverse transcription products after incubation with dNTP mix (upper panels in Fig. 5A). No increase was observed when primers specific for early DNA were used or when dNTPs were omitted from the reaction. Cytoplasmic complexes isolated at 5 h post-infection displayed a 1.6-fold increase of late reverse transcription products after ERT (bottom panel in Fig. 5A). This decrease is likely due to maturation of the cRTCs during the first 5 h of infection, although the differences in ERT activity between the 2 h and 5 h complexes did not reach statistical significance. Because of low concentration of nRTCs isolated at 2 h post-infection, we were unable to measure ERT in this population of complexes. However, as shown in the bottom panels of Fig. 5A, nRTCs isolated at 5 h post-infection did carry out reverse transcription, although rather inefficiently compared to cytoplasmic complexes (approximately 1.3-fold increase in late reverse transcription products). These findings, together with immunoprecipitation data (Fig. 4), suggest that some complexes may complete reverse transcription in the nucleus. Since there is much more HIV-specific complexes in the cytoplasm than in the nucleus (Figs. 1, 2, 3), it appears that most cytoplasmic complexes detected by PCR with primers specific for early HIV-1 DNA did not complete reverse transcription, suggesting that only a small portion of early RTCs are capable of completing their maturation and staying on the pathway to integration.

### *In vitro *integration of HIV-1 PICs into isolated chromatin

To compare integrative capacity of cytoplasmic and nuclear complexes, and to evaluate the effect of ERT on integration, we analyzed *in vitro *integration of the complexes into immunoprecipitated chromatin. Since previous studies demonstrated significance of nucleosomal organization of the chromatin for HIV-1 integration [27,28]., we used immunoprecipitated chromatin, rather than naked DNA, as a target for integration.

Cytoplasmic and nuclear complexes, subjected to ERT in the absence (control) or presence of dNTPs, were incubated with chromatin in the presence of 0.25 mM ATP for 1 h at 37°C. Integration of HIV-1 DNA was analyzed by *Alu*-LTR-based real-time nested-PCR according to [29]. Integrative capacity of cytoplasmic complexes isolated at 2 h post-infection increased two-fold after the ERT reaction (Fig. 5B). Analysis of nuclear complexes at 2 h p.i. was not performed due to miniscule amounts of viral complexes in the nucleus at this time point. Complexes isolated from cytoplasm at 5 h post-infection showed a 1.25-fold increase of integration after ERT. The increase in integration correlated with results of the ERT reaction (Fig. 5A), indicating that *in vitro *completion of RT reaction in cRTCs increased their ability to integrate into chromatin. ERT did not increase the integrative capacity of nRTCs isolated at 5 h post-infection (Fig. 5B), although the low rate of ERT was observed in these complexes (Fig. 5A).

Without ERT, cytoplasmic and nuclear complexes purified at 5 h post-infection appeared to have similar integration capacities (Fig. 5B). A decrease in integration of nPICs after ERT may be due to inhibition by dNTPs [30]. This inhibition should also affect integration of cytoplasmic complexes, but in this case it is not seen due to an increase in integration efficiency because of ERT. This result indicates that cytoplasmic and nuclear complexes (PICs) have a similar integration capacity despite differences in their bulk protein composition (e.g., lack of p24 and decreased amount of RT in nPICs, Fig. 4), consistent with a notion that only a small fraction of cytoplasmic and nuclear RTCs represents the integration-competent PICs. Our data also suggest, that completion of reverse transcription in a small part of nRTCs containing incomplete reverse transcripts does not appear to contribute to integration.

## Conclusion

Taken together, results presented in this report show that most HIV-1 RTCs complete reverse transcription in the cytoplasm and then translocate into the nucleus. Completion of the reverse transcription correlates with changes in protein composition of the RTCs which may contribute to the ability of complexes to translocate through the nuclear pore complex. However, in dividing cells, some RTCs can get into the nuclear compartment during the mitosis before completing DNA synthesis. Thus, population of nRTCs is heterogeneous, with some complexes containing incomplete reverse transcription products and RT, similar to cRTCs. These nRTCs are capable of reverse transcription, indicating that their maturation may potentially continue in the nuclear compartment. Nevertheless, this process appears to be rather inefficient and does not seem to significantly contribute to the amount of integration-competent complexes, suggesting that maturation of RTCs and their conversion into PICs is completed in the cytoplasm. This study adds to HIV-1 RTC/PIC characterization and advances our understanding of RTC maturation.

## Methods

### Cells and viruses

HEK 293T and HeLa cells were purchased from ATCC (Manassas, VA). Cells were maintained at 37°C in atmosphere containing 5% CO_2 _in Dulbecco's modified Eagle medium (DMEM) supplemented with 2 mM glutamine, 10% (v/v) fetal bovine serum (Bio Whittaker), 100 units/ml penicillin, and 100 units/ml streptomycin. CEM cells (ATCC CCL-119) used for chromatin isolation were grown in RPMI-1640 containing 2 mM glutamine, 10% (v/v) FBS, 100 units/ml penicillin, and 100 units/ml streptomycin. To generate replication-incompetent HIV-1 vectors for infection of HeLa cells, HEK 293T cells were seeded in 75 cm^2 ^flasks and cultivated up to approximately 70% monolayer. Then cells were co-transfected using Metafectene (Biontex) with NLHXB [31] or the GFP-expressing NL43GFP11 [15] molecular clones and a vector encoding the Env protein of the amphotropic MLV, pcDNA-Env(MLV) (provided by Dr. N. Landau). 72 h after transfection recombinant virus particles were harvested, filtered through a 0.45-μm-pore-size filter and incubated for 1 h at 37°C in a buffer containing 10 mM MgCl_2 _and 60 U/ml of RNase-free DNase I (Roche, Indianapolis, IN). Virus particles were concentrated from the culture media by centrifugation through a 30% sucrose cushion in PBS at 24,000 RPM in a Beckman SW-28 rotor for 2 h at 4°C. Virus pellets were resuspended in Dulbecco's modified Eagle medium containing 20 mM HEPES (pH 7.4). For infection, viral titers were normalized by p24 ELISA (PerkinElmer Life Sciences, Boston, MA) to 0.5 pg of p24 per cell. Infection of HeLa cells was performed in 6-well plates by spinoculation at 18°C (to prevent viral internalization by the cells during spinoculation) according to a published protocol).)[14]. After spinoculation virus-containing media was removed, cells were washed twice with pre-warmed PBS and 1% FBS and incubated at 37°C for 2, 5 or 24 h.

### Synchronization of cells and cell cycle analysis

HeLa cells were synchronized in the G1/S phase as described previously [32]. Briefly, cells were cultivated in DMEM with 10% fetal bovine serum to 50% confluence, then 2 mM of thymidine (Sigma, St. Louis, MO) was added. After 16 h, cells were washed with pre-warmed PBS and 1% FBS and infected as described above. Cell cycle distribution was analyzed by flow cytometry (FACS Calibur, Becton-Dickinson, Mountain View, CA) essentially as described previously [33].

### Cell fractionation, RTC isolation and purification of RNA/DNA

Approximately 2 × 10^7 ^infected HeLa cells were harvested using Trypsin (0.5 g/L) in10 mM EDTA and washed with 80 ml cold PBS twice. Fractionation of cells and isolation of the RTCs was performed essentially as described by Fassati and Goff [3] with several modifications. Hypotonic buffer for preparation of the cytoplasm was supplemented with 0.025% Brij 96 to disrupt RTC association with the cytoskeleton. Nuclei before homogenization were washed from components of cytoplasm with 0.5% Triton X-100 in isotonic buffer for 5 min on ice, vortexed for 10 seconds and precipitated by low-speed centrifugation. The nuclear pellets were washed twice with isotonic buffer and additionally separated from cytoplasmic components by centrifugation through density gradient of Iodixanol as described by Graham et al. [34]. After subsequent wash in isotonic buffer nuclei were homogenized using EZ-Grind kit (G Biosciences, St. Louis, MO).

Viral RTCs were purified from cytoplasmic and nuclear extracts by centrifugation through a 45% sucrose cushion (in hypotonic buffer for cytoplasmic and in isotonic buffer for nuclear extracts) at 34,000 RPM (100,000 × g) in a Beckman SW-60 rotor for 3 h at 4°C. Pellets of HIV-1 RTCs from cytoplasmic and nuclear fractions were resuspended in 200 μl of buffer K (20 mM HEPES, pH 7.3, 150 mM KCl, 5 mM MgCl_2_, 1 mM dithiothreitol, and 1 tablet of Complete Mini EDTA-free protease inhibitor cocktail [Roche] per 10 ml) [35], snap-frozen in liquid N_2_, and stored at -80°C.

### Immunoprecipitation of RTCs

RTCs were immunoprecipitated from suspensions of purified cytoplasmic and nuclear complexes according to [36]. Suspensions were diluted by buffer K, aliquoted into 200 μl samples and incubated for 2 h at 4°C with 4 μl of non-immune rabbit or mouse serum (Sigma) and 2.5 μg of protein G-Sepharose 4 Fast Flow (Amersham Biosciences, Piscataway, NJ) in buffer K containing 1% bovine serum albumin (BSA) and 1 mg/ml salmon sperm DNA (5 Prime-3 Prime, Boulder, CO). Protein G-bound complexes were pelleted (5000 × g) and clarified supernatants were reacted with 4 μg of each of the following antibodies: mouse monoclonal antibodies for MA, RT and IN (ABI, Columbia, MD), CA [37] and PML (Santa Cruz Biotechnology, Santa Cruz, CA); rabbit polyclonal antibodies to Vpr (a kind gift from Josephine Sire) and Ini1 (Santa Cruz Biotechnology), and purified mouse and rabbit IgG (Jackson's Laboratories) as isotype controls. After an overnight incubation at 4°C, 2.5 μg of protein G-Sepharose was added and incubation continued for an additional 2 h. Protein G-bound immune complexes were pelleted and washed three times with buffer K supplemented with 0.1% Triton X-100, and washed once without Triton X-100. DNA was isolated from immune precipitates and analyzed by real-time PCR. DNA values immunoprecipitated by isotype control were subtracted from the data obtained with corresponding specific antibody.

### Purification of HIV-1-specific nucleic acids and RT reaction

RNA was purified from suspensions of cPICs and nPICs using RNA STAT-50LS RNA isolation solution (Tel-Test, Friendswood, TX) according to manufacturer's protocol. DNA was purified from suspensions of RTCs mixed with 5 μg of glycogen using IsoQuick DNA Isolation kit (ORCA, Bothell, WA). Reverse transcription of isolated RNA to cDNA for subsequent real-time PCR analysis was performed using GeneAmp RNA PCR Kit components (Applied Biosystems, Foster City, CA) according to manufacturer's protocol.

### PCR analysis

Primers specific for mitochondrial DNA (forward primer, Mito1: 5'-GAA TGT CTG CAC AGC CAC TT-3'; reverse primer, Mito2: 5'-AGA AAG GCT AGG ACC AAA CC-3') were used to assess contamination of the nuclear fraction with cytoplasmic components. DNA from purified viral RTCs was analyzed by regular and real-time PCR using primers M667 (5'-GGCTAACTAGGGAACCCACTG-3') and AA55 (5'-CTGCTAGAGATTTTCCACACTGAC-3') specific for the negative-strand "strong-stop" DNA (the early reverse transcription product), and FOR-LATE (5'-TGTGTGCCCGTCTGTTGTGT-3') and REV-LATE-NL43 (5'-GAGTCCTGCGTCGAGAGATC-3') specific for the late reverse transcription products [38]. Real-time PCR was performed in triplicate using iQ SYBR Green Supermix Kit (BioRad, Hercules, CA) and fluorescence was measured on CFD 3200 Opticon System. Serial dilutions of DNA from 8E5 cells (CEM cell line containing a single copy of HIV-1 LAV provirus per cell) were used as the quantitative standards [39].

### Endogenous reverse transcription

Complexes were incubated with or without dNTP mix (2 mM) for 4 h at 37°C in ERT buffer (100 mM Tris-HCl, pH 8.0; 15 mM NaCl; 5 mM MgCl_2_; 1 mM DTT), and ERT products were analyzed by real-time PCR with primers specific for early (a control) and late HIV-1 DNA.

### Chromatin isolation

Chromatin was isolated from CEM cells as described previously [40] with following modifications. Following fixation with 1% formaldehyde cells were lysed with buffer containing 1% SDS, 10 mM EDTA, 50 mM Tris-HCl, pH 8.1, sonicated to reduce DNA length to 200–1,000 bp, and debris was removed by centrifugation. The chromatin solution was pre-cleared on protein G beads pre-adsorbed with sonicated salmon sperm DNA to minimize non-specific binding and then incubated with a mixture of antibodies against histone H3 phosphorylated on serine 10 (Upstate Cell Signaling Solutions), Pol II (Santa Cruz) and 2,2,7 trimethyl-guanosine (Oncogene) overnight at 4°C. Immune complexes were collected using protein G beads pre-adsorbed with sonicated salmon sperm DNA.

## Competing interests

The author(s) declare that they have no competing interests.

## Authors' contributions

SI carried out RTC purification and analysis, immunoprecipitation of RTCs, FACS analysis, endogenous RT and integration assays, and participated in drafting the manuscript. RB carried out chromatin immunoprecipitation. MA participated in RTC purification and isolation of HIV-1 DNA. FK participated in the design of the study and contributed to drafting of the manuscript. MB conceived of the study, participated in its design and coordination and drafted the manuscript. All authors read and approved the final manuscript.
